# Regulation of p53 by the 14-3-3 protein interaction network: new opportunities for drug discovery in cancer

**DOI:** 10.1038/s41420-020-00362-3

**Published:** 2020-11-16

**Authors:** Marta Falcicchio, Jake A. Ward, Salvador Macip, Richard G. Doveston

**Affiliations:** 1 Leicester Institute for Structural and Chemical Biology, University of Leicester, University Road, Leicester, LE1 7RH UK; 2 School of Chemistry, University of Leicester, University Road, Leicester, LE1 7RH UK; 3 Mechanisms of Cancer and Ageing Lab, Department of Molecular and Cell Biology, University of Leicester, University Road, Leicester, LE1 7RH UK; 4 FoodLab, Faculty of Health Sciences, Universitat Oberta de Catalunya, Barcelona, Spain

**Keywords:** Tumour-suppressor proteins, Tumour-suppressor proteins

## Abstract

Most cancers evolve to disable the p53 pathway, a key tumour suppressor mechanism that prevents transformation and malignant cell growth. However, only ~50% exhibit inactivating mutations of p53, while in the rest its activity is suppressed by changes in the proteins that modulate the pathway. Therefore, restoring p53 activity in cells in which it is still wild type is a highly attractive therapeutic strategy that could be effective in many different cancer types. To this end, drugs can be used to stabilise p53 levels by modulating its regulatory pathways. However, despite the emergence of promising strategies, drug development has stalled in clinical trials. The need for alternative approaches has shifted the spotlight to the 14-3-3 family of proteins, which strongly influence p53 stability and transcriptional activity through direct and indirect interactions. Here, we present the first detailed review of how 14-3-3 proteins regulate p53, with special emphasis on the mechanisms involved in their binding to different members of the pathway. This information will be important to design new compounds that can reactivate p53 in cancer cells by influencing protein–protein interactions. The intricate relationship between the 14-3-3 isoforms and the p53 pathway suggests that many potential drug targets for p53 reactivation could be identified and exploited to design novel antineoplastic therapies with a wide range of applications.

## Facts

- 14-3-3 proteins play diverse and important roles in the regulation of wild-type p53 activity.
- 14-3-3σ has distinctive roles and engages in non-canonical interactions with partner proteins.
- Selective modulation of 14-3-3 protein–protein interactions, via stabilisation or inhibition, is a new avenue toward p53 activation and antineoplastic drug development.

## Open questions

- What is the true extent of 14-3-3 protein involvement in p53 regulation and tumour suppression?
- Can the currently putative links between 14-3-3 activity and p53 function be firmly established?
- What is the basis of 14-3-3 isoform-specific function and can this be harnessed for selective targeting?
- There is an urgent need for molecular tools that facilitate more detailed and expansive studies into the role of 14-3-3 in p53 activation.

## Introduction: 14-3-3 proteins as master regulators of p53 activity

**Fig. 1:**
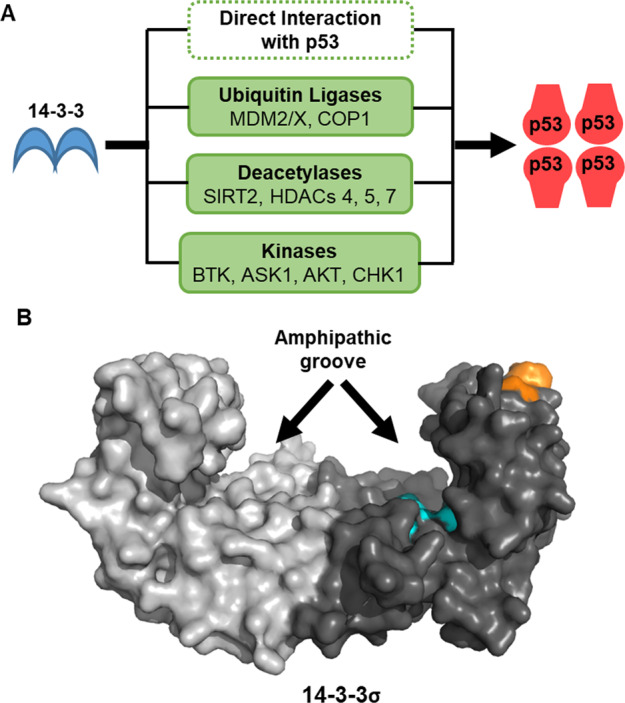
14-3-3 Regulation of p53. **a** Summary of the 14-3-3 PPIs involved in upstream p53 regulation. **b** Crystal structure of dimeric 14-3-3σ. For one monomer, the canonical phosphate binding site within the amphipathic groove (cyan) and the 14-3-3σ-specific secondary binding site (orange). PDB: 1YWT.

The transcription factor p53 is one of the most important tumour suppressor proteins^1^. It is inactivated in many cancer types, either through mutation or through disruption of its regulatory mechanisms^2^. p53 functions are modulated by a vast number of post-translational modifications and interacting proteins^3,4^. Among these, the 14-3-3 family of hub proteins play important roles in p53 regulation. Notably, 14-3-3 proteins regulate many processes that occur downstream following p53 activation in response to DNA damage (Fig. 1a)^5^.

14-3-3 proteins are a family of dimeric adapter proteins with seven isoforms in humans (β, γ, ε, η, σ, τ, ζ). They interact with over 200 partner proteins to modulate their enzymatic activity, subcellular localisation, or ability to interact with other proteins^6^. In this way, 14-3-3 proteins have a prominent role in cell cycle regulation^5^ and are frequently implicated in cancer progression^7^. 14-3-3 protein–protein interactions (PPI) are typically phosphorylation dependent. Recognition motifs usually lie within disordered domains of partner proteins that contain phosphorylated serine or threonine residues. These bind to an amphipathic groove that contains a basic pocket formed by L49, R56, R129 and R127, which is conserved across the 14-3-3 family (Fig. 1b)^5^. There are three consensus 14-3-3 recognition motifs: mode I [RSX(pS/T)XP], mode II [RX(Y/F)X(pS/T)XP] or mode III [pS/TX-COOH]. However, the presence of a + 2 proline residue only occurs in approximately 50% of known mode I/II interactions^8^. Furthermore, there are notable examples that deviate from the consensus motifs. For example, 14-3-3σ binding to the C-terminus of p53 via the C-terminal motif FKpTEGPDSD-COOH (vide infra)^9^. Due to their dimeric nature, 14-3-3 proteins can interact with two phosphorylation sites within the same protein in a bivalent fashion (typically 15-20 residues apart). Each 14-3-3 monomer can also interact with distinct partner proteins independently of each other^10^. 14-3-3 PPIs can be inhibited or stabilised by small molecules and peptides but thus far these are not selective for specific PPIs or 14-3-3 isoforms^6^.

In this review, we will systematically summarise all the potential mechanisms (experimentally confirmed or putative) by which the different members of the 14-3-3 family of proteins could regulate p53 activity. This is not limited to the direct effects of the 14-3-3/p53 interaction but also include indirect effects trough p53 ubiquitination (by regulation of MDM2, MDMX and COP1 (constitutive photomorphogenesis 1)), acetylation (by regulation of SIRT2, and HDAC4, 5 and 7) and phosphorylation (by regulation of CHK1, BTK, ASK1 and AKT). All this information together supports the emerging view that 14-3-3 proteins are master regulators of p53 activity by simultaneously impinging on different elements of the pathway. This makes them especially attractive targets for designing new drugs to reactivate p53 in the context of cancer.

## Direct Interactions between p53 and 14-3-3 proteins

p53 does not contain a classical 14-3-3-binding motif but there is overwhelming evidence of a direct interaction between the two proteins that influences p53 activity. 14-3-3γ, 14-3-3ε, 14-3-3ζ, 14-3-3τ and 14-3-3σ have all been shown to regulate p53 through direct interactions. The role of 14-3-3σ (and to an extent 14-3-3τ) is very distinct from that played by the other isoforms. No interaction of p53 with 14-3-3β or 14-3-3η has been yet identified. Intriguingly, the 14-3-3/p53 PPI can be modulated by drug-like molecules which makes it an attractive drug target^11^.

### p53 interactions with 14-3-3γ, 14-3-3ε and 14-3-3ζ

#### 14-3-3 binding mode

**Fig. 2:**
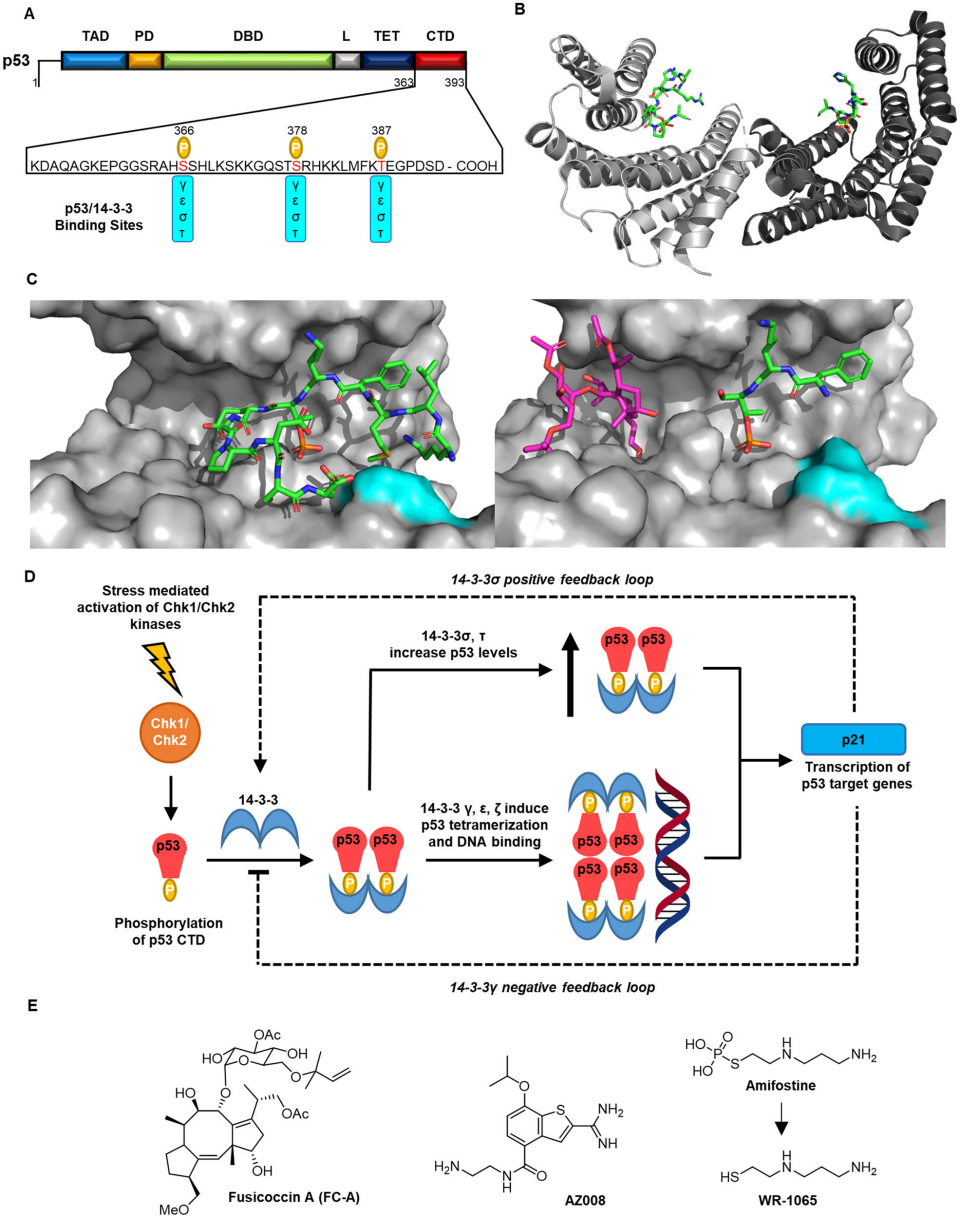
Direct Interactions between p53 and 14-3-3 proteins. **a** The structure of p53 indicating 14-3-3-binding sites. CTD C-terminal domain, TAD transcriptional activation domain, PD proline domain, DBD DNA-binding domain, L linker region, TET tetramerisation domain, P phosphorylation. **b** Crystal structure of dimeric 14-3-3σ (grey) complexed with a p53pT387 12mer peptide (green). PDB: 5MOC. **c** Left: Crystal structure showing the p53pT387 12mer peptide (green) bound to the 14-3-3σ amphipathic groove (grey). 14-3-3σ R60 is shown in cyan. PDB = 5MOC. Right: Crystal structure of the ternary complex of 14-3-3σ (grey), p53pT387 (green) and FC-A (purple). 14-3-3σ R60 is shown in cyan. PDB = 5MXO. **d** The regulation of p53 by 14-3-3 proteins. **e** Structure of small molecules stabilisers of 14-3-3σ/p53 interaction.

14-3-3γ and 14-3-3ε proteins interact with the disordered C-terminal domain (CTD) of p53 in a phosphorylation-dependent manner. The structure of p53 and the 14-3-3-binding motifs are shown in Fig. 2a. Binding of the two isoforms was demonstrated through pull-down assays and IP and blotting using U2OS osteosarcoma cells^12^. After DNA damage, several p53 CTD residues are phosphorylated by Chk1/2 kinase in response to ATM activation^13^. Of these, phosphorylation of S378 with simultaneous dephosphorylation of S376 was proposed to be the critical p53 post-translational modifications required for 14-3-3 binding (Fig. 2a)^14^. However, the post-translational events controlling this interplay are complex. Transfection of a p53 construct with mutation of S376 to alanine (S376A) into U2OS osteosarcoma cells abrogated 14-3-3 binding. Surprisingly, the corresponding mutant S378A had a less pronounced effect, indicating that phosphorylation of S376 is an important regulatory event^12^. Two other potential phosphorylated 14-3-3-binding sites were identified in a peptide affinity screen^15^. In fluorescence polarisation (FP) and analytical ultracentrifugation (AU) assays phosphorylated peptides mimicking the motifs around S366 and T387 were also found to have notable binding affinities to 14-3-3γ and 14-3-3ε. These were comparable to that for the motif around pS378 (Table 1). The corresponding phosphopeptide mimicking the S376 motif did not show a significant affinity for these 14-3-3 isoforms. Diphosphorylated p53 peptides were found to have 50–100 times higher affinity for 14-3-3γ and 14-3-3ε, highlighting the significance of bivalent interactions made possible by the dimeric nature of 14-3-3 (ref. ^15^). Phosphorylated, tetrameric p53 CTD constructs also had higher 14-3-3 affinities (10×) compared to the monomeric peptides^15^. The relevance of all three p53 phosphorylation sites (S366, S378 and T387) was demonstrated through a series of in vitro pull-down assays and IP experiments using the lysates of H1299 cells^16^. Interestingly, alanine point mutations to any single phosphorylation site did not affect 14-3-3γ and 14-3-3ε binding in vitro and in cells, but mutation of all three completely abrogated binding. This points to a ‘fail safe’ mechanism that regulates 14-3-3γ and 14-3-3ε binding to p53, whereby no single phosphorylation event is essential^16^.

**Table 1:** p53 CTD phosphopeptides affinity to 14-3-3 isoforms as determined by FP assay.

p53 CTD peptides	*K*_d_ (μM)^a^			
	*γ*	*ε*	*τ*	*σ*
pS366	17 ± 2	16 ± 2	28 ± 3	24 ± 4
pS378	20 ± 2	18 ± 3	27 ± 3	22 ± 3
pT387	14 ± 3	11 ± 2	24 ± 4	23 ± 3
pS366/pT387	0.14 ± 0.07	0.18 ± 0.05	6.2 ± 0.3	3.5 ± 0.4
pS366 /pS378	0.48 ± 0.05	0.^51^ ± 0.03	2.1 ± 0.4	2.2 ± 0.3
pS378/pT387	0.45 ± 0.03	0.80 ± 0.06	3.3 ± 0.2	7.3 ± 0.3
wt (362–393)	n.q.	n.q.	n.q.	n.q.

*n.q.* not quantifiable.

^a^Values taken from published data^16^

14-3-3ζ was shown to interact with p53 through immunoprecipitation (IP) and western blotting (WB) experiments conducted using the lysates of co-transfected HEK293 cells^17^. Interestingly, the interaction is perturbed by PKA-mediated phosphorylation of 14-3-3ζ which impairs 14-3-3 dimerisation^17^. Investigations to determine where p53 interacts with 14-3-3ζ were not conducted, but it likely binds in an analogous fashion to 14-3-3γ and 14-3-3ε.

#### Role in p53 regulation

Interaction of 14-3-3γ, ε and ζ isoforms increases p53 transcriptional activity (Fig. 2d). Co-transfection of p53 with 14-3-3γ or 14-3-3ε into H1299 cells increased p21 transcription (p21 is a p53 target gene) by ~2.5-fold as shown using a p21 luciferase reporter assay^16^. In line with the 14-3-3-binding experiments, transfection of p53 constructs bearing point mutations to any single 14-3-3γ- and 14-3-3ε-binding phosphorylation site (S366, S378, T387) did not affect this increased transcriptional response. However, transfection of a construct with alanine mutations to all three sites showed no increase in p21 transcription compared to a control experiment run in the absence of 14-3-3γ and ε isoforms^16^. These data are consistent with those obtained from other p21 reporter experiments conducted in Saos2 cells expressing endogenous 14-3-3. Transfection of these cells with p53 constructs bearing the point mutations S376A and S378A did not result in any change to p21 transcription compared to wt p53 (ref. ^12^). Co-transfection of p53 and 14-3-3ζ into HEK293 cells also led to an increase in p53 transcriptional activity as shown in a p21 luciferase reporter assay^17^. In a manner consistent with the respective binding study, when 14-3-3ζ phosphorylation was inhibited using H89, a PKA inhibitor, p21 transcription was further increased. This provides further evidence pointing to the importance of 14-3-3ζ dimerisation^17^.

The increased transcriptional activity of p53 induced by interactions with 14-3-3γ, ε and ζ is a result of enhanced p53 binding to DNA. There is no evidence to suggest they contribute to increased p53 levels in cells. The role of 14-3-3 in mediating p53-DNA binding was first demonstrated through an electrophoretic mobility shift assay (EMSA) and antibody capture experiments^14,16^. These showed that binding of recombinant p53 to a ^32^P-labelled sequence-specific DNA construct was specifically induced by the addition of GST-tagged 14-3-3ζ protein in a PKA-dependent manner^14^. The same DNA binding was not observed for a p53 S378A mutant, or when a p53 CTD specific antibody was present, providing further confirmation of 14-3-3ζ binding to phosphorylated S378 within the p53 CTD^14^.

Similar observations were made for 14-3-3γ and 14-3-3ε through further EMSA experiments in combination with FP. Recombinant p53, phosphorylated by Chk1/2 kinases, was incubated with sequence specific DNA representing a p21 response element. It was found that DNA bound to a p53/14-3-3 binary complex in a 14-3-3γ and 14-3-3ε concentration-dependent manner^15,16^. The affinity of p53 to DNA was determined using fluorescently labelled p21 response element DNA in an FP binding assay. The binding affinity was found to be increased by a factor of ~2× in the presence of 14-3-3γ (*K*_d_: 44 to 22 nM) and 14-3-3ε (*K*_d_: 48 to 25 nM)^16^. Using these data, and with further evidence from AU experiments, Fersht et al.^15^ proposed a mechanism by which 14-3-3 promote p53 binding to DNA. The evidence suggests that 14-3-3γ and 14-3-3ε sequester dimeric p53 and induce tetramerisation, with two 14-3-3 dimers binding to a single p53 tetramer. p53 tetramers have a higher affinity for DNA compared to the dimeric form. This is interesting because the stoichiometry indicates 1:1 binding of p53 monomer to 14-3-3 monomer. It is thus not suggestive of a bivalent interaction between a single diphosphorylated p53 monomer and a 14-3-3 dimer which the peptide studies indicated was favourable in terms of binding affinity^15^.

As common in many biological pathways, a negative feedback loop controls p53–14-3-3 dynamics (Fig. 2d). For example, p53 is known to down-regulate the expression of 14-3-3γ^18^ and has even been shown to mediate its degradation by the proteasome^19^.

### p53 Interactions with 14-3-3σ and 14-3-3τ

14-3-3σ and τ interact with p53 in a manner that is distinct from the other isoforms and the interaction has a different regulatory role. Furthermore, p53 activation upregulates 14-3-3σ expression in a positive feedback loop (Fig. 2d)^20,21^, which contrasts with the 14-3-3γ isoform.

#### 14-3-3 binding mode

p53 was shown to interact with 14-3-3σ in co-IP experiments using the lysates of A549 cells. In these experiments, p53 was activated by adriamycin- and irradiation-induced DNA damage^21^. Further evidence for these interactions was also obtained in an analogous experiment using H1299 cells expressing inducible wt p53^16^. Here, p53 was activated by camptothecin-induced DNA damage. As with the 14-3-3γ, ε and ζ isoforms, p53 phosphorylation of S366, S378 and T387 was observed by IP (Fig. 2a)^16^. The binding affinities of 14-3-3σ and 14-3-3τ to peptides mimicking the p53 motifs around S366, S378 and T387 were determined by FP^16^. These were found to be only slightly reduced compared to the 14-3-3γ, ε and ζ isoforms. Again, diphosphorylated peptides had much higher affinities.

The interaction of 14-3-3σ with the p53 phosphorylated T387 motif with 14-3-3σ has received the most focus. This is despite the physiological significance of this phosphorylation site being unclear. The affinity of the p53 T387 phosphopeptide motif to 14-3-3σ was determined to have a *K*_d_ = 16.3 ± 0.7 μM using isothermal titration calorimetry (ITC)^9^. This was consistent with *K*_d_ obtained using FP (23 ± 3 µM)^16^. Significantly, a protein X-ray crystallography structure of a phosphopeptide mimicking the p53 motif around T387 in complex with 14-3-3σ gives detailed structural insight (Fig. 2b, c)^9^. The p53 peptide interacts with the 14-3-3 binding groove via a unique turn conformation induced by G389 and P390 that allows the C-terminus to form a salt bridge interaction with R60 of 14-3-3σ^9^. Subsequently, the dynamics of this interaction have been studied in greater detail^22^. The propensity of the peptide motif to adopt this conformation is an important factor that determines binding affinity as shown by a combination of ITC, surface plasmon resonance (SPR), NMR and molecular dynamics (MD) simulations^22^.

Intriguingly, there is compelling evidence to show that 14-3-3σ interacts with p53 via a secondary binding site in a phosphorylation-independent manner (Fig. 2a). Using R1B/L17 cells co-transfected with p53 and Flag-14-3-3σ constructs it was found that the CTD of 14-3-3σ (aa 153-248) interacted with p53 most efficiently compared to wt or N-terminal constructs^21^. This result was confirmed in an in vitro GST pull-down experiment^21^. It is an interesting observation because the conserved 14-3-3 residues essential for phosphopeptide binding (K49, K120, R56, and R127) were not present in the 14-3-3σ CTD construct. Complementary to this, IP experiments in H1299 cells showed that 14-3-3σ and 14-3-3τ interacted with p53 when any or even all of the three putative phosphorylation sites (S366, S378, T387) were mutated to alanine^16^. The interaction was however dependent on camptothecin-induced DNA damage. This suggests that p53 activation is required for 14-3-3σ and 14-3-3τ binding but that this occurs via an altogether different mechanism. Thus, these data imply two things:

(1) Activation of p53 in response to DNA damage is essential for 14-3-3σ binding but occurs via an unknown mechanism.

(2) Activated p53 interacts with 14-3-3σ via a phosphorylation-independent binding mode that involves a hitherto poorly understood 14-3-3σ secondary binding site.

#### Role in p53 regulation

Like the other isoforms, 14-3-3σ increases the transcriptional activity of p53. In both R1B/L17 and mouse embryonic fibroblast (MEF) cells co-transfection of 14-3-3σ led to the activation of a p53 luciferase reporter gene in a dose-dependent manner. No such effect was seen when a reporter gene containing mutated p53 binding sites was used^21^. Thus, this provides strong evidence that the effect of 14-3-3σ is directly related to p53 activity. 14-3-3σ was also shown to increase p21 gene expression in R1B/L17 cells transfected with 14-3-3σ, as shown by northern blot analysis^21^. Furthermore, co-transfection of p53 with 14-3-3σ into H1299 cells increased p21 transcription by ~2.5 fold in a luciferase reporter assay^16^. In agreement with the binding studies, mutation of all three key p53 phosphorylation sites did not abrogate the effect on p21 transcriptional activity^16^. Thus, these data provide further evidence for 14-3-3σ engaging in a non-canonical interaction.

The consequence of the 14-3-3σ/p53 PPI on p53 transcription are mainly attributed to an increase in cellular p53 levels, but there is also evidence to suggest it mediates p53 oligomerisation. In two studies, IP experiments have shown that 14-3-3σ expression in H1299 cells leads to increased levels of p53^16,21^. To support this, immunofluorescence staining of RB/L17 cells showed that co-transfection of 14-3-3σ markedly increased p53 levels compared to an endogenous system. The increased levels of p53 result from greater stability and a longer half-life. This was demonstrated by monitoring ^35^S-labelled p53 in an IP time course experiment^21^. Enhanced cellular stability of p53 has been directly attributed to the inhibition of its degradation by MDM2 (ref. ^21^). It is not clear if the interaction of 14-3-3σ has a role in protecting p53 from MDM2 ubiquitination, or if this is primarily a result of a direct interaction between 14-3-3σ and MDM2 (vide infra). 14-3-3σ mediated oligomerisation of p53 has been shown by glutaraldehyde crosslinking followed by SDS-PAGE analysis. This finding was corroborated in pull-down experiments using GFP-labelled p53 and immobilised p53 in the presence or absence of 14-3-3σ^21^. However, no increase in DNA binding was observed using the experiments previously described^16^.

### Small-molecule stabilisation of the 14-3-3/p53 PPI

Stabilising the interaction of p53 with 14-3-3 proteins, and especially 14-3-3σ, should have an anti-proliferative effect based on the experimental data reviewed above. It is therefore of interest in the context of antineoplastic therapies to find and develop molecular glues that can enhance this PPI. The turn conformation adopted by the p53 CTD phosphorylated T387-binding motif reveals a ligand-binding pocket that corresponds to that occupied by FC-A and other known 14-3-3 stabilisers^9^. Thus, it has been the focus of drug discovery efforts although, as highlighted above, the biological relevance of this motif is unclear. There are three known stabilisers of the interaction between 14-3-3σ and the p53 T387 phosphopeptide motif (Fig. 2e).

*Fusicoccin A***:** FC-A induces a modest four-fold stabilisation of the interaction as shown by ITC and FP experiments^23^. An X-ray crystal structure of FC-A bound to the 14-3-3σ–p53 complex was obtained by crystal soaking (Fig. 2c). However, addition of the stabiliser yielded a crystal structure with a highly disordered peptide-binding partner. This is likely an artefact of crystal soaking but could also point to an atypical FC-A mode of action.

*AZ-008***:** AZ-008 was discovered as a weak stabiliser of this PPI during a fragment-based drug discovery campaign^24^. FP and SPR experiments indicated that the compound has a reproducible two-fold stabilisation effect. NMR studies provided strong evidence that the molecule binds in the amphipathic binding groove corroborated the stabilising effect. Unfortunately, a crystal structure could not be obtained.

*Amifostine***:** Amifostine is a radioprotective prodrug approved by the FDA for the treatment of head and neck cancer. In vivo, it is hydrolysed to the active species WR-1065 (ref. ^25^). Treatment of IEC-6 cells with amifostine led to increased p53 levels, transcriptional activity and nuclear localisation. It was also shown to inhibit MDM2-mediated degradation of p53 (ref. ^26^). These observations are all consistent with the role of 14-3-3σ and thus suggest that amifostine stabilises its interaction with p53. Proximity ligation experiments confirmed this, showing that amifostine induced proximity of the two proteins in the nucleus. Currently, there is no molecular explanation for these observations. Independently of 14-3-3σ, WR-1065 has been shown to directly bind and activate p53 through a JNK-dependent signalling pathway, which lead to an increase of p53-response genes levels^25,27^. Mutation of p53 C173, C235 or C239 to serine significantly reduced p53-DNA binding and its transcriptional activity^28^. Formation of a covalent disulfide bond between WR-1065 and p53 might underpin the molecular mechanism behind p53 activation by amifostine^25,27^.

## 14-3-3 regulation of p53 ubiquitination

Ubiquitination plays a fundamental role in regulating the stability and cellular localisation of p53. Ubiquitination is a multi-step enzymatic process that involves three key enzymes—E1 ubiquitin-activating enzyme, E2 ubiquitin-conjugating enzyme and E3 ubiquitin-ligating enzyme^29^. The result is the addition of one or more ubiquitin moieties (mono- or poly-ubiquitination, respectively) to key lysine residues on target proteins^29^. In healthy and unstressed cells, p53 is maintained at low levels because it undergoes rapid degradation by the ubiquitin-mediated proteasome^30^. The in vivo half-life of p53 is reported to be <20 min^31,32^. In particular, E3 ubiquitin ligase proteins play a crucial role in the regulation of p53 degradation, cellular localisation and transcription^3,33,34^. To date, the E3 ubiquitin ligases known to target p53 are: MDM2, COP1, Pirh2, TRIM69, TRIM59, UBE2T, RBCK1, SMYD3, ARF-BP1, CHIP, WWP1, MSL2 and E4F1 (ref. ^3^). The principle negative regulator for p53 is Mouse Double Minute 2 Protein (MDM2). A close homologue of MDM2, MDMX, also plays an important role but does not possess E3 ubiquitin ligase activity. Through direct interactions, 14-3-3 proteins control the activities of MDM2, MDMX and COP1 to regulate the p53 ubiquitination landscape.

### 14-3-3σ regulation of MDM2

All seven 14-3-3 isoforms directly interact with MDM2 to influence its regulation of p53. Of all the 14-3-3 isoforms, the biological implications of the MDM2/14-3-3σ PPI are distinctive.

#### 14-3-3σ binding mode

The interaction of 14-3-3σ with MDM2 is phosphorylation dependent and reliant on the CTD of the ubiquitin ligase (amino acids: 440–491, Fig. 3a). This was shown through co-IP experiments from lysates of HEK293T cells transfected with wild type and truncated MDM2 (amino acids 1–439) constructs^35^. To date, the specific phosphorylation sites on MDM2, and the responsible kinase(s), have not been defined. However, the two requirements for binding point to phosphorylation of the MDM2 CTD. The MDM2 CTD is known to be phosphorylated by ATM kinase at S395, S407, S386, T419, S425 and S429 (ref. ^36^). However, these residues were present in the truncated MDM2 construct (1–439) which did not bind to 14-3-3σ. Therefore, this suggests that these phosphorylation sites are not implicated in 14-3-3σ binding. Based on these data, two hypotheses can be proposed:

**Fig. 3:**
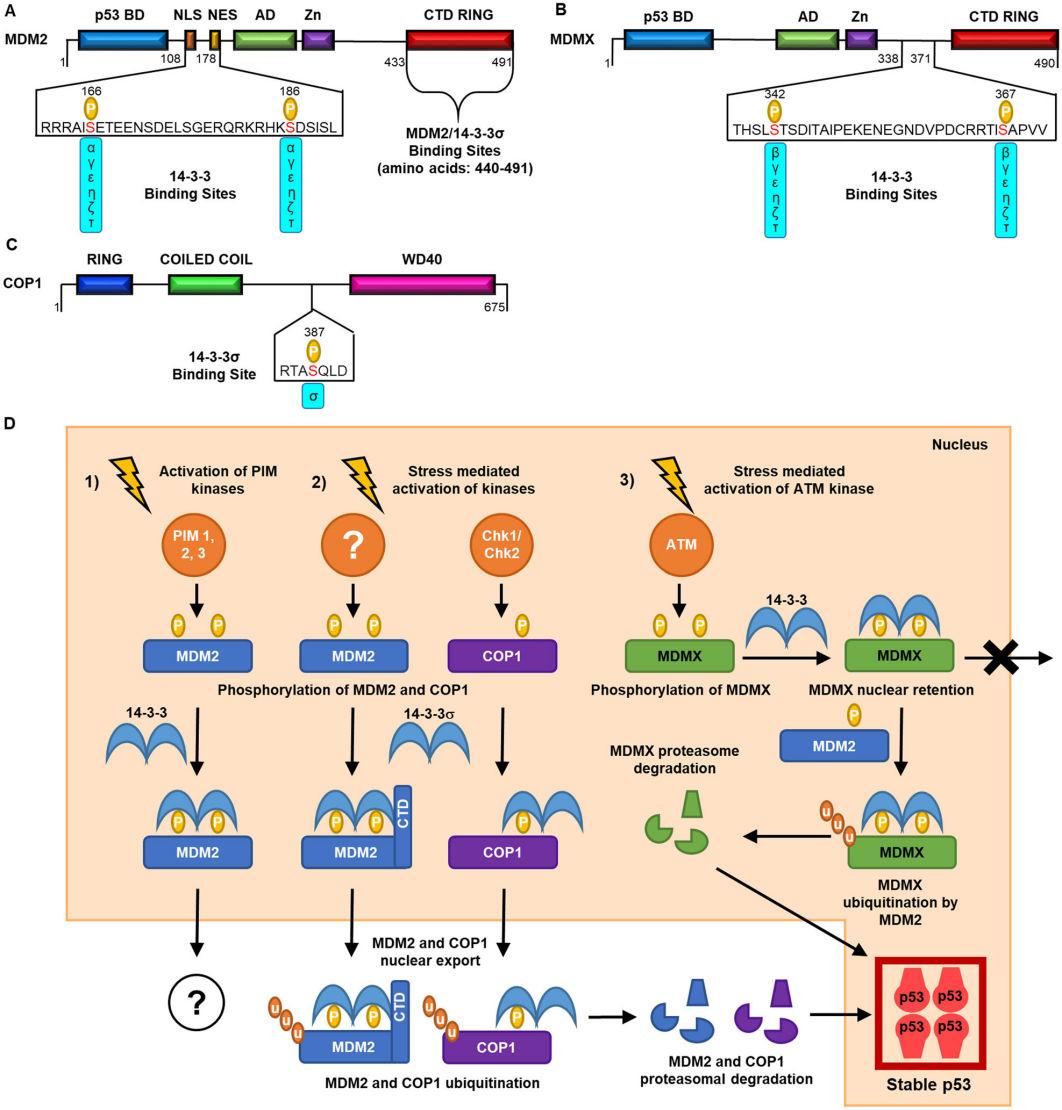
The regulation of p53 ubiquitination by 14-3-3 proteins. **a** The structure of MDM2. **b** The structure of MDMX. **c** The structure of COP1, all indicating 14-3-3-binding sites. NLS nuclear localisation signal, NES nuclear export signal, p53 PD p53-binding domain, AD acidic domain, Zn zinc domain, CTD RING C-terminal RING domain, P phosphorylation. **d** The roles of 14-3-3 PPIs with MDM2, MDMX and COP1 in p53 regulation.

(1) 14-3-3σ binding is entirely phosphorylation dependent but truncation of the CTD has a negative effect on kinase activity or protein conformation. The respective phosphorylation site(s) could be the ATM targets in the CTD, although they not do not fall within typical 14-3-3-binding motifs. Other potential sites may also lie in the NLS/NES of MDM2 in a manner consistent with experimental data for the other 14-3-3 isoforms (vide infra).

(2) 14-3-3σ binding is dependent on phosphorylation and a secondary phosphorylation-*independent* interaction with the CTD of MDM2. This mechanism might correspond to the non-canonical binding mode seen for the 14-3-3σ/p53 PPI.

The second hypothesis is supported by convincing evidence. The C-terminus section of 14-3-3σ (amino acids 153-248) bound to MDM2 as efficiently as the full-length 14-3-3σ construct^35^. Again, the conserved 14-3-3σ residues essential for phosphopeptide binding (K49, K120, R56 and R127) were not present in this 14-3-3σ construct. Together, these data might imply that an interaction between the aforementioned proposed secondary binding site of 14-3-3σ and the CTD of MDM2 is an important feature of this PPI that conveys 14-3-3σ isoform specificity.

#### Role of the 14-3-3σ/MDM2 PPI in p53 regulation

14-3-3σ binding negatively regulates MDM2 ubiquitination activity and influences its cellular localisation (Fig. 3d)^35^. As a direct consequence of this, p53 levels are stabilised and its transcriptional activity is increased^21^. 14-3-3σ conveys this effect by facilitating the auto-ubiquitination of MDM2 as shown in HEK293T cells through transfection of 14-3-3σ^35^. Furthermore, it was demonstrated that HCT116 cells overexpressing 14-3-3σ had a higher MDM2 turn-over rate compared to 14-3-3σ deficient cells. 14-3-3σ also negatively regulates MDM2 activity in HEK293T and HCT116 cells by promoting its nuclear exclusion (Fig. 3d)^35^. Consequently, shuttling of MDM2 into the cytoplasm antagonises the p53 nuclear export activity of MDM2^35^.

The significance of the 14-3-3–MDM2 interaction has been further demonstrated in tumorigenesis models^35,37^. Rat1-Akt cells with constitutive PKB/Akt activity, a known positive regulator of MDM2, were transfected with 14-3-3σ^35^. The addition of 14-3-3σ resulted in an increase in p53 levels and expression of p53 target gene PUMA^35^. Furthermore, this led to a significantly reduced tumour volume in nude mice, which correlated with reduced expression of MDM2 (refs. ^35,37^). The constitutive PKB/Akt activity is an important consideration in this experiment. MDM2 is known to be phosphorylated by PKB/Akt at S166 and S186/S188 within the NLS/NES^38,39^. PKB/Akt is not known to facilitate MDM2 binding to the other 14-3-3 isoforms. This is intriguing because these PPIs are reliant on phosphorylation of the same residues (vide infra)^40^. Although there are many complex cellular processes to consider, it is another nuance that points to isoform specificity.

#### 14-3-3α γ, ε, ζ, η and τ binding mode

The remaining six 14-3-3 isoforms (α γ, ε, ζ, η and τ) bind MDM2 following its phosphorylation at residues S166 and S186 by Pro-viral Integration Site for Moloney Murine Leukaemia Virus (PIM) kinases 1, 2 and 3, as shown through a series of in vitro IP and WB experiments (Fig. 3a)^40^. S166 and S186 lie within the NLS/NES of MDM2. Alanine mutations at either residue S166 or S186 markedly reduced 14-3-3 binding and thus both are required for optimal affinity. This suggests that MDM2 engages in a bivalent interaction with dimeric 14-3-3. In contrast to 14-3-3σ, the MDM2 C-terminal RING-finger domain did not have any influence on binding to these 14-3-3 isoforms^40^, another indication of potential isoform specificity.

MDM2 is also phosphorylated by PKB/Akt within the NLS/NES at S166 (corresponds to PIM phosphorylation) and S188 (+2 relative to the PIM site) (Fig. 3a). This modification is known to positively regulate MDM2 by inhibiting its self-ubiquitination activity and translocation to the nucleus^39–42^. PKB/Akt phosphorylation is often implicated in 14-3-3 PPIs^43,44^ and its activity is not detrimental to 14-3-3σ binding in vivo (vide supra). However, its phosphorylation of S166 and S188 does not facilitate 14-3-3 binding according to the available in vitro data. Subsequent phosphorylation of S186 by PIM is not abrogated by this PKB/Akt activity. This may be a further indication that a bivalent interaction involving MDM2 phosphorylation of S186 and S186 is critical.

#### Putative role of the MDM2/14-3-3α γ, ε, ζ, η, τ PPIs in p53 regulation

The cellular implications of the 14-3-3 α γ, ε, ζ, η and τ isoform interactions with MDM2 have not been explored. PIM kinases are known to be positive regulators of MDM2 activity and their overexpression is associated with an oncogenic impact on the p53 pathway^45^. Given the tendency for the interactions of these isoforms to be pro-survival, it is logical to propose they work in synergy with PIM to positively regulate MDM2 and promote p53 degradation. 14-3-3σ would appear to have a distinctive role as p53 protectorate and thus the potential for isoform specificity is worthy of further investigation.

### 14-3-3 regulation of MDMX

Mouse double minute X protein (MDMX) is a close homologue of MDM2 that also negatively regulates p53 (refs. ^46–48^). However, MDMX does not possess E3 ubiquitin ligase activity^49,50^. MDMX stabilises MDM2 via a direct interaction between their Really Interesting New Gene (RING) domains^51,52^. The resultant MDM2–MDMX hetero-complex is more effective at ubiquitinating p53 than the MDM2 homodimer and MDMX–MDM2 hetero-dimerisation is shown to be essential for p53 poly-ubiquitination^53–56^. The MDM2/MDMX PPI has a prominent role in p53 regulation^55,57^.

#### 14-3-3 binding mode

All 14-3-3 isoforms, apart from 14-3-3σ, interact with MDMX in a phosphorylation-dependent manner as shown by a combination of cell-based mass spectrometry^58^, IP and WB experiments^55,59^. The absence of any interaction with 14-3-3σ is reported consistently across the studies reviewed here. 14-3-3 binding occurs in response to DNA damage^60^ and is reliant on phosphorylation at S342 by Chk2 (ref. ^61^) and AMPK^62^; and S367 by Chk1/2 (refs. ^58,59^) and Akt (Fig. 3b)^55^. These residues are located in a disordered linker region of MDMX between the Zn^2+^ domain and the C-terminal RING domain. MDMX is also phosphorylated at S391 and S403, but these are not implicated in 14-3-3 binding^59^. Mutation of 14-3-3γ K50, which is essential for interacting with phosphate moieties, abrogates MDMX binding in co-transfected HEK293 cells^58^. Furthermore, the competitive 14-3-3 inhibitor difopein inhibits the interaction^55^ which is also a strong indication of classical phosphorylation-dependent 14-3-3 binding.

MDMX S367 lies within a classical 14-3-3 mode 1/2-binding motif (DCRRTIpSAPVV) and is the most important phosphorylation site for its interaction with 14-3-3 (Fig. 3b). This has been demonstrated in a number of binding studies whereby mutation of this residue abrogates 14-3-3 binding in a number of cell lines: HEK293 (refs. ^55,58^), H129 (refs. ^59,62^) and wt mouse embryo fibroblasts^62^. These observations have been confirmed by proteomic analyses^55^. In addition, synthetic phosphopeptides that mimic the S367-binding motif have been used to perform in vitro pull-down experiments^59^ and displacement assays^58^ that corroborate the cell data. P369 is also an important element of the 14-3-3 consensus motif, and mutation of this residue leads to a reduced interaction in cellular binding experiments^58,59^.

MDMX S342 appears to play a secondary role in MDMX binding to 14-3-3. One study conducted using H1299 cells transfected with a MDMX S342A mutant showed that this had no effect on 14-3-3 binding. However, subsequent reports indicate that mutation of S342 has a detrimental effect on the PPI in both H1299 (ref. ^62^) and MCF-7 cells^61^. S342 does not lie within a classical 14-3-3-binding motif however (KLTHSLpSTSDI, Fig. 3b). The fact that two MDMX phosphorylation sites 25 amino acids apart are important for optimal binding is suggestive a bivalent interaction between one MDMX protein and dimeric 14-3-3. Evidence to support this theory has been obtained by a pull-down experiment whereby a dimerisation-defective 14-3-3 mutant did not show any interaction with MDMX in transfected U2OS cells^61^.

#### Role of the 14-3-3/MDMX PPI in p53 regulation

14-3-3 binding to MDMX increases the transcriptional activity of p53 in luciferase reporter models where MDM2 expression is stable (Fig. 3d). Co-transfection of 14-3-3τ and MDMX in HCT116 cells significantly restored p53 transcriptional activity that was diminished when cells were transfected with MDMX alone^59^. This restorative effect was lost when a MDMX S367A mutant was transfected, providing further evidence that the 14-3-3τ binding to MDMX was a critical factor. Repression of p53 transcription was also observed in H1299 and MEFs expressing MDMX S367A^60^. When H1299 cells were transfected with 14-3-3γ, p53 transcriptional was again enhanced^58^. In this model, transfection of a 14-3-3γ K50E mutant did not induce this effect. Although this evidence does not specifically implicate MDMX, it provides further indication that a 14-3-3 PPI is requisite.

The 14-3-3/MDMX interaction leads to increased cellular levels of p53 (Fig. 3d). When U2OS cells were transfected with MDMX, WB experiments confirmed that cellular levels of p53 and p21 diminished as expected. This effect was reversed when 14-3-3γ was also transfected, providing a strong indication that its interaction with MDMX is important for p53 stability^58^. Indeed, ectopic expression of 14-3-3γ was shown to reverse MDMX-enhanced p53 ubiquitination in H1299 cells^58^, and no 14-3-3γ binding to MDM2 was detectable in this model. It suggests that 14-3-3γ protects p53 from proteasomal degradation through its interaction with MDMX.

14-3-3 promotes MDMX nuclear accumulation (Fig. 3d). As a direct consequence, p53 cellular levels and transcriptional activity are elevated. MDMX translocates to the nucleus by interacting with MDM2, or independently in response to DNA damage (Fig. 3d)^59^. There is also conflicting evidence that suggests non-phosphorylated MDMX accumulates in the nucleus, where phosphorylation occurs and promotes nuclear export^61^. Therefore, here we refer simply to MDMX nuclear ‘accumulation’ rather than specifically implying translocation. Immunofluorescence staining of transfected U2OS cells showed that the MDMX S367A mutant does not accumulate in the nucleus in response to DNA damage^59,61^. Furthermore, co-transfection with YFP-difopein prevented nuclear accumulation of wt MDMX^61^. These observations are consistent with the binding data. They provide strong evidence that a phosphorylation-dependent interaction with one or more 14-3-3 isoforms plays an important role in promoting the nuclear accumulation of MDMX. The precise mechanism by which 14-3-3σ facilitates this process is not clear. 14-3-3 binding to MDMX in the cytoplasm could cause a conformational change that reveals a ‘cryptic’ MDMX nuclear localisation sequence and thus promotes nuclear import^59^. Alternatively, 14-3-3 could bind to phosphorylated MDMX in the nucleus to prevent its nuclear export^61^.

14-3-3τ promotes the degradation of phosphorylated MDMX (Fig. 3d). In MCF-7 cells, expression of 14-3-3τ mutants that cannot bind to phosphorylated partners resulted in stabilisation of MDMX levels, as shown by WB analysis. Furthermore, degradation of MDMX was prevented upon introduction of the 14-3-3 inhibitor YFP-difopein^61^. In a similar experiment, transfection of 14-3-3τ in U2OS cells enhanced MDMX degradation^59^. Phosphorylation of MDMX S367 is important for effective MDMX ubiquitination and thus proteasomal degradation^59^. Ubiquitin Ni-NTA pull-down experiments and WB analysis performed using transfected HCT116 (ref. ^59^) and H1299 cells^60^ have shown that the MDMX S367A mutant is resistant to MDM2-mediated ubiquitination. Therefore, this is a strong indication that 14-3-3-mediated degradation of MDMX is a result of enhanced ubiquitination. One hypothetical explanation for this is that 14-3-3 proteins prevent the deubiquitinating enzyme HAUSP from interacting with MDMX^59,61^.

### 14-3-3 regulation of COP1

COP1 is another example of a p53-targeting E3 ubiquitin ligase protein that is regulated by 14-3-3 proteins. In this case, an interaction between COP1 and only one 14-3-3 isoform, 14-3-3σ, has been reported^63–65^.

#### 14-3-3σ binding mode

In response to DNA damage, COP1 is phosphorylated by ATM kinase at S387 (ref. ^66^). S387 is located within a motif (RTApSQL) that shows many similarities to consensus 14-3-3-binding motifs, but lacks the +2 proline residue (Fig. 3c)^63^. Phosphorylation of S367 is essential for 14-3-3σ binding as shown by co-IP experiments in co-transfected 283 T cells. Transfection of the COP1 S367A mutant abolished the interaction while the introduction of phosphatase inhibitors increased the level of binding. These experiments also showed that the N-terminal region of 14-3-3σ (amino acids: 1–161) was required for binding to COP1, but the C-terminal region (amino acids: 153–248) was not. This is a clear indication that the interaction occurs via a classical, phosphorylation-dependent mechanism and not via the putative 14-3-3σ secondary binding site discussed previously. Subsequently, in vitro GST pull-down experiments were used to pin-point the 14-3-3σ interaction site on COP1. As expected, a construct of COP1 containing S367 (amino acids: 216–420) showed strong interaction with 14-3-3σ. Interestingly, the N-terminus of COP1 (amino acids: 1–226) also showed a weak interaction with 14-3-3σ^64^. This perhaps indicates that a secondary interaction is also involved.

#### Role of the 14-3-3σ/COP1 PPI in p53 regulation

The 14-3-3σ/COP1 interaction stabilises p53 levels in cells (Fig. 3d). Immunoblotting experiments conducted using co-transfected 293T cells have shown that the introduction of 14-3-3σ rescues COP1 induced depletion of p53 levels in a dose-dependent manner^64^. This observation is consistent with IP/WB experiments in H1299 cells that show 14-3-3σ antagonises COP1-mediated p53 ubiquitination^64^ and nuclear export (Fig. 3d)^63^. Importantly, these effects are also observed in MDM2 null MEF cells, indicating that they are independent of MDM2 activity.

p53 transcriptional activity is increased by the 14-3-3σ/COP1 interaction. qPCR and a p53 luciferase reported assay have shown that p53 gene expression is increased upon ectopic expression of 14-3-3σ in HCT116 and U2OS cells overexpressing COP1 (ref. ^64^). This effect was compromised in COP1-depleted cells, thus providing further evidence that this PPI directly influences p53 transcriptional activity^64^.

Presumably, as a result of p53 upregulation, 14-3-3σ impairs the proliferation and survival of HCT116 cells overexpressing COP1 (refs. ^63,64^). Fluorescence-associated cell sorting analysis showed that a higher percentage of HCT116 cells expressing 14-3-3σ arrested in the G phase compared to cells not expressing 14-3-3σ^64^. Furthermore, the injection of an adenovirus containing 14-3-3σ directly into mouse xenograft tumours (tumours comprised of HCT116 cells overexpressing COP1) reduced growth rate^64^.

14-3-3σ downregulates COP1 levels in response to DNA damage by enhancing COP1 ubiquitination activity in HCT116 and 293T cells (Fig. 3d)^63,64^. 14-3-3σ protein reduced COP1 protein levels in 293T cells in a dose-dependent manner, an effect that was absent in cells expressing mutant COP1 (S387A)^63^. Ubiquitination assays were used to demonstrate that the level of polyubiquitinated COP1 was higher in stressed 293T and HCT116 cells following co-transfection with 14-3-3σ compared with 14-3-3σ null cells^63,64^. Furthermore, an in vitro ubiquitination assay to demonstrate that COP1 self-ubiquitination activity was enhanced in the presence of 14-3-3σ protein^64^.

COP1 dynamically shuttles between the nucleus and the cytoplasm to regulate its target proteins and in response to DNA damage COP1 is excluded from the nucleus^63,66^. Time-lapse confocal microscopy was used to monitor the cellular localisation of fluorescently labelled COP1 (refs. ^63,66^). 14-3-3σ regulates the cellular localisation of COP1 in response to DNA damage by antagonising the dynamic shuttling of COP1, sequestering it in the cytoplasm (Fig. 3d)^63^. 14-3-3σ contains an NES which is essential for mediating the nuclear exclusion of COP1 (ref. ^63^). The mutation of key residues in the NES region of 14-3-3σ (I205A and L208A) negatively impacted on the ability of bound 14-3-3σ to regulate the nuclear export of COP1 (ref. ^63^). As a result, the COP1-mediated nuclear export of p53 is inhibited by 14-3-3σ^63^. In HCT116 cells, the short hairpin RNA (shRNA) knockdown of 14-3-3σ antagonises the DNA damage-induced nuclear exclusion of COP1 (ref. ^63^).

14-3-3σ is itself a substrate for COP1 and the ubiquitination process is mediated by the COP9 signalosome subunit 6 (CSN6)^67^. CSN6 binds to 14-3-3σ at the CTD (amino acids: 153–248) which leads to the recruitment of COP1 and subsequent degradation of 14-3-3σ^67^. In addition, CSN6 also antagonises COP1 self-ubiquitination, resulting in COP1 stabilisation^67^. Deregulation of the CSN6-COP1 axis can promote tumorigenesis^67^. Therefore, targeting the CSN6/COP1 or CSN6/14-3-3σ through PPI inhibition is a potential novel therapeutic strategy in targeting cancers driven by CSN6-COP1 overexpression.

## 14-3-3 regulation of p53 acetylation

Acetylation of p53 in response to cellular stress results in protein stabilisation, sequence-specific association with DNA and transcriptional activation^68–74^. p53 is acetylated by p300/CBP acetyltransferase at key lysine residues predominantly clustered around the CTD (K164, K370, K372, K373, K381, K382 and K386)^68,69^. Acetylation of K120 by members of the MYST HAT family (TIP60, MOF and MOZ) is responsible for the transcription of pro-apoptotic factors PUMA and BAX, but not cell cycle arrest genes^74,75^. In addition, the acetylation of p53 at K320 by p300/CBP-associated factor in response to DNA damage results in the activation of genes to promote cell survival but not apoptosis^69,72^.

The acetylation of p53 can be reversed by various histone deacetylases (HDACs). In humans, there are four classes of HDAC (Class I, Class IIa/b, Class III and Class IV)^76^. These classes can be divided into two families based on the conserved deacetylase domain and their specific cofactors: the HDAC family (Class I and Class IIa/b) and the sirtuin protein family (Class III and Class IV)^76^. HDAC 1 (Class I) is one of the most prominent deacetylase enzymes for p53 and it represses p53-dependent transcriptional activation, cell cycle arrest and apoptosis^77^. There are numerous other deacetylases which differentially regulate p53, including HDAC8 (Class I), HDAC 4, 5 and 7 (Class IIa), HDAC6 (Class IIb), SIRTs 1, 2 and 3 (Class III)^77–80^. The role of acetylation and deacetylation in p53 function has been reviewed previously^3,81^. 14-3-3 proteins play an important role in the regulation of SIRT2, a class III HDAC, with a direct impact on p53 activity. They also regulate class IIa HDACS 4, 5 and 7 but there is no evidence of a direct consequence for p53 activity in these cases. However, their effect on HDAC activity is significant and it is highly probable these interactions influence p53 behaviour.

### 14-3-3 regulation of SIRT2

#### 14-3-3 binding mode

14-3-3β and 14-3-3γ bind to SIRT2 in a phosphorylation-dependent manner^82^. These interactions were identified using IP and WB experiments conducted in HEK293 cells expressing wt AKT. When a kinase deficient AKT construct was expressed the interaction was lost. No interaction between SIRT2 and 14-3-3ζ or 14-3-3ε was observed and 14-3-3σ was not investigated. The molecular details of the interaction between 14-3-3β and 14-3-3γ have not been established. SIRT2 is known to be phosphorylated at S368 by CDK1, which plays a role in the regulation of cell proliferation^83^. However, S368 is not located in a classical 14-3-3 motif. This site lacks a positively charged residue in the −3, −4 or −5 position relative to S368 and also the proline residue is in the +1 position, rather than +2 position, which rarely occurs in 14-3-3-binding motifs^8^. Other potential sites for phosphorylation are present, but there is no experimental evidence to suggest they have any physiological relevance. Therefore, the exact site of SIRT2 involved in 14-3-3 interaction requires further investigation.

#### Role of the 14-3-3/SIRT2 PPI in p53 regulation

**Fig. 4:**
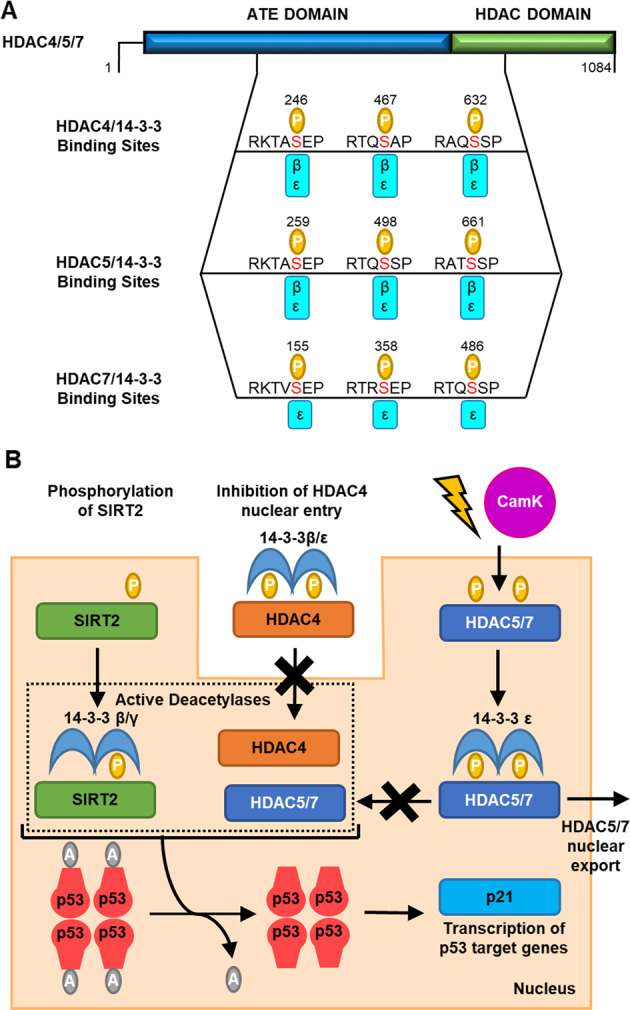
The regulation of p53 acetylation by 14-3-3 proteins. **a** The general structure for HDACs 4, 5 and 7 indicating 14-3-3-binding sites. The 14-3-3-binding site on SIRT2 is not known. ATE amino terminus extension. **b** The roles or putative roles of 14-3-3 interactions with SIRT2 and HDACs 4, 5 and 7 in p53 regulation.

SIRT2 deacetylates p53 to repress its transcriptional activity^82,84–86^. 14-3-3β and 14-3-3γ promote SIRT2 deacetylation of p53 (Fig. 4b)^82^. In addition, 14-3-3β and 14-3-3γ have been shown to augment SIRT2-mediated inhibition of p53 transcriptional activity (Fig. 4b). In a p21 luciferase reporter assay conducted in transfected HEK293T cells, expression of SIRT2 decreased transcription of p21 and p53 reporter genes. This inhibitory effect was potentiated by the addition of 14-3-3β and 14-3-3γ but not 14-3-3ε or 14-3-3ζ isoforms. This effect was lost when nicotinamide, an SIRT2 inhibitor was introduced. Together, this suggests that 14-3-3β and 14-3-3γ play a direct role in SIRT2 regulation of p53 transcriptional activity^82^. However, it does not rule out 14-3-3 conveying these effects via alternative mechanisms.

### 14-3-3 regulation of HDAC4

#### 14-3-3 binding mode

14-3-3 protein binds to HDAC4 in a phosphorylation-dependent manner as shown by IP and WB analysis^87–89^. 14-3-3 isoform-specific antibodies were used to identify 14-3-3β and 14-3-3ε as the isoforms that interact with phosphorylated HDAC4 (ref. ^87^). The treatment of TAg Jurkat and NIH 3T3 cells with the serine/threonine kinase inhibitor staurosporine resulted in reduced interaction between HDAC4 and 14-3-3β/ε^87^ Conversely, under hyperphosphorylation conditions induced by treatment of cells with phosphatase inhibitor calyculin A, there was an increase in the formation of HDAC4/14-3-3 complexes^87^.

There are three key serine residues in HDAC4 (S246, S467 and S632) that are phosphorylated by calcium/calmodulin dependent kinases I or II (CamK I/II) to facilitate binding of 14-3-3β and 14-3-3ε^87^. These phosphorylation sites resemble but do not completely match, classical mode 1 14-3-3-binding motifs (S246: LRKTApSEPNL; S467: LGRTQpSAPLP; S632: LSRAQpSSPAS; Fig. 4a). The mutation of all three key serine residues of HDAC4 (S246A, S467A and S632A) abrogated binding to 14-3-3β and 14-3-3ε, whereas mutation of anyone residue had no effect on the binding of 14-3-3β and 14-3-3ε. A double mutation of (S246A, S467A) considerably reduced binding, perhaps pointing to a bivalent interaction involving unusually distant phosphorylation sites^87^. Further investigation into the molecular aspects of this PPI is required in order to understand the binding mechanism in more detail.

#### Putative role of the 14-3-3/HDAC4 PPI in p53 regulation

To date, direct evidence linking the 14-3-3/HDAC4 PPI to p53 regulation is lacking. In response to DNA damage HDAC4 is recruited to the nucleus where it associates with p53 at G_2_/M promoters and represses transcription of pro-survival genes such as the cyclins^78^. 14-3-3β and 14-3-3ε have been shown to sequester HDAC4 in the cytoplasm where it is unable to associate with p53. Thus 14-3-3 is likely to inhibit HDAC4 deacetylation of p53 and promote transcription of cell survival genes (Fig. 4b)^87^. Immunofluorescence was used to monitor the cellular localisation of RFP-HDAC4 in U20S cells, in the presence and absence of 14-3-3β^87^. Transfection of 14-3-3β increased the proportion of cells with predominantly cytoplasmic RFP-HDAC4 (ref. ^87^). In addition, the expression of the HDAC4 triple mutant (S246A, S467A and S632A), which is unable to bind 14-3-3β/ε, impaired HDAC4 cytoplasmic localisation^87^. Similar results were also obtained in an analogous study^89^. The binding of 14-3-3β/ε antagonises the ability of HDAC4 to bind importin α, potentially by masking the nuclear localisation sequence, which is key for translocation into the nucleus^87^.

### **14-3-3 regulation of HDAC5**

#### 14-3-3 binding mode

14-3-3β and 14-3-3ε also bind to HDAC5 in a phosphorylation-dependent manner as shown by IP and WB experiments^87,89^. The binding of 14-3-3β and 14-3-3ε to HDAC5 involves the phosphorylation of S259 and S498 by CamK in response to genotoxic stress (Fig. 4a)^89^. As with HDAC4, these resemble mode 1 14-3-3-binding motifs (S259: LRKTApSEPNL; S498: LSRTQpSSPLP) (Fig. 4a). A HDAC5 mutant lacking amino acids 248–615 was unable to bind to 14-3-3ε and simultaneous double mutation of HDAC5 (S259A and S498A) led to complete loss of 14-3-3ε binding^89^, whereas a single mutation of either S259A or S498A of HDAC5 had no effect on the binding of 14-3-3 protein. Thus, binding occurs via a bivalent interaction between distal amino acids like that seen for HDAC4, or two distinct monovalent interactions.

#### Putative role of the 14-3-3/HDAC5 PPI in p53 regulation

Again, a direct link between this PPI and p53 regulation has not been demonstrated but there is strong evidence that suggests its involvement. The deacetylation of K120 of p53 by HDAC5 is known to regulate p53-mediated transactivation in response to genotoxic stress^79^. Nuclear exclusion of HDAC5 results in increased levels of acetylated p53 at K120 and recruitment to pro-apoptotic genes (Fig. 4b)^79^. Binding of 14-3-3ε to HDAC5 leads to its nuclear export^79,89,90^. It is therefore reasonable to assume that the 14-3-3/HDAC5 PPI promotes transcription of pro-apoptotic p53 target genes. In COS cells, the cytoplasmic and nuclear levels of HDAC5 are unaffected by co-expression of 14-3-3ε protein, with HDAC5 predominately located in the nucleus as shown by immunofluorescence assays^89^. However, activation of the CamK signalling pathway promoted the association of HDAC5 with 14-3-3ε and resulted in their co-localisation within the cytoplasm as shown by immunofluorescence assay^89^. As with HDAC4, the binding of HDAC5 to 14-3-3β and 14-3-3ε masks its nuclear localisation sequence and antagonises its binding to importin α^87^. In HCT116 cells, the expression of a HDAC5 S259A-S498A double mutant abrogated the acetylation of p53 at K120 in response to prolonged genotoxic stress. This led to cellular senescence rather than apoptosis in control HCT166 cells^79^. Although this does not explicitly implicate the involvement of 14-3-3 proteins, it is remarkable that these two residues are also essential for the interaction between HDAC5 and 14-3-3. It is likely that 14-3-3 binding to HDAC5 also abrogates acetylation of p53 K120.

### 14-3-3 regulation of HDAC7

#### 14-3-3 binding mode

The interaction of mouse HDAC7 with 14-3-3ε protein involves phosphorylation of three key serine residues by CamK I: S178, S349 and S479 (ref. ^91^). Mutation of all three serine residues significantly reduced the affinity of HDAC7 for 14-3-3ε^91^ Single mutation of S178A had a greater impact on the binding affinity of 14-3-3ε than a single mutation of S344A or S479A thus pointing to a monovalent interaction^91^. The three serine residues lie within sites that closely resemble classical mode 1 14-3-3-binding motifs (Fig. 4a). In human HDAC7 there are three 14-3-3-binding motifs that closely resemble those in mouse HDAC7, these are residues S155, S358, S486 (motifs S155: LRKTVpSEPNL; S358: LSRTRpSEPLPP; S486: LSRAQpSSPAA, Fig. 4a). To date, the binding of human HDAC7 and 14-3-3 has not been investigated.

#### Putative role of the 14-3-3/HDAC7 PPI in p53 regulation

Currently, there is no evidence to confirm that this PPI directly regulates p53 activity. HDAC7 plays a role in inducing apoptosis via deacetylation of p53 L382^92^. 14-3-3ε protein regulates the stability of HDAC7^93^. Ectopic expression of CamK I or 14-3-3ε in HEK293 cells increased levels of HDAC7, and the co-expression of both proteins resulted in additive accumulation^94^. Therefore, it is possible that 14-3-3 binding to HDAC7 upregulates p53 deacetylation and transcriptional activity (Fig. 4b). However, 14-3-3ε protein also regulates the cellular localisation of HDAC7, by promoting its cytoplasmic accumulation^94^. This was demonstrated using immunofluorescence microscopy to study the cellular localisation of wt and single, double and triple HDAC7 mutant proteins (S178A and/or S344A and/or S479A) in NIH3T3 cells. They found that while wild-type HDAC7 accumulated in the cytoplasm, expression of the mutated HDAC7 (single, double and triple mutants) with reduced 14-3-3ε binding affinity accumulated in the nucleus^91^. This effect was most apparent following triple mutation of HDAC7 (S178A, S344A and S479A). This would suggest that 14-3-3 negatively regulates p53 transcriptional activity.

### Indirect 14-3-3 regulation of p53 acetylation via MDM2

MDM2-mediated ubiquitination of the p53 CTD inhibits p53 acetylation by p300/CBP acetyltransferase to antagonises p53 activation^95,96^. Furthermore, MDM2 recruits HDAC1 to p53 to promote p53 deacetylation^96^. Therefore, given the key role that 14-3-3 protein plays in regulating MDM2 stability and association with p53, it is considered likely that 14-3-3 indirectly regulates acetylation of p53 via MDM2. However, the evidence to confirm this link directly is currently lacking.

## 14-3-3 regulation of p53 phosphorylation

The phosphorylation and subsequent dephosphorylation of p53 at multiple serine and threonine residues is important in regulating its stability and activation^3^. The phosphorylation sites of p53 are predominately clustered in the N-terminal domain (S6, S9, S15, T18, S20, S33, S37, S46, T55 and T81) and the C-terminal linker and basic regions (S315, S366, S371, S376, S378, T387 and S392)^13,97,98^. The majority of p53 phosphorylation occurs in response to DNA damage and results in p53 stabilisation and activation. Notably, phosphorylation of N-terminal residues by ATM, ATR, DNA-PK, Chk1 and Chk2 kinases have been shown to be important in alleviating MDM2-mediated p53 degradation^97,99–101^. However, it is important to note that there is evidence that cast doubts about the significance and relevance of phosphorylation of certain N-terminal residues of p53. For example, it has been reported that certain amino acids (S6, S9, S15, S33, S315, S392 and T18), and even pairs of amino acids, had no role in p53 stabilisation following actinomycin treatment or UV and γ irradiation. In addition, some in vivo data in mice showed that mutation of p53 at S18 or S23 led to only subtle changes in p53 function^102–105^. The dephosphorylation of p53 residues can revert p53 activation^106^. Together, this points to a refined but complex mechanism of p53 regulation through phosphorylation^107^.

14-3-3 proteins regulate p53 activity via a direct interaction with phosphorylated p53 (vide supra). In addition, they interact with the p53-targeting kinases Chk1, BTK, ASK1, p38, Jnk and AKT to regulate their activity. Although no direct link between these 14-3-3 PPIs and p53 regulation has been established, the evidence suggests that 14-3-3 proteins likely have a significant influence on p53 activity in this way.

### 14-3-3 regulation of Chk1

In response to DNA damage, Chk1 activates p53 by phosphorylating of serine/threonine residues predominantly located in the p53 N-terminal domain (S15, T18, S20 and S37)^13,108,109^. Chk1 itself is activated by phosphorylation of S317 and S345 by ATR kinase^110,111^.

#### 14-3-3 binding mode

**Fig. 5:**
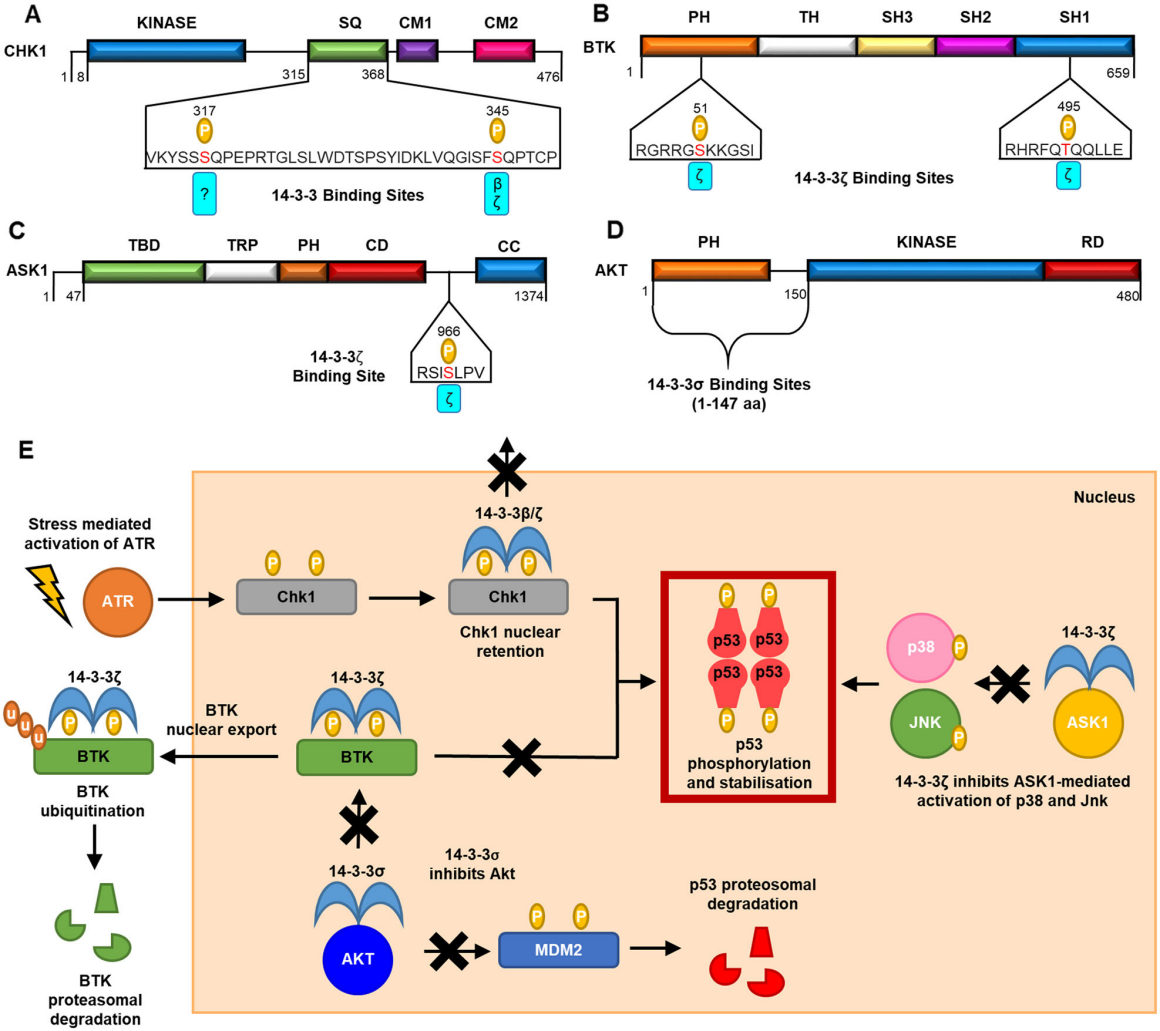
The regulation of p53 phosphorylation by 14-3-3 proteins. The structures of p53-associated kinases: **a** Chk1, **b** BTK, **c** ASK1 and **d** AKT, all indicating 14-3-3-binding sites. SQ serine/glycine cluster, CM conserved motif, PH pleckstrin homology domain, TH Tec homology domain, SH Src-homology domain, TBD TRX-binding domain, TRP tetratricopeptide repeats domain, CD catalytic domain, CC coiled-coil region, RD regulatory domain. **e** The putative roles of 14-3-3 interactions with Chk1, BTK, ASK1 and AKT in p53 regulation.

14-3-3β, 14-3-3ζ and 14-3-3σ interact with phosphorylated Chk1. 14-3-3β and 14-3-3ζ binding was demonstrated through pull-down experiments conducted using GST-labelled 14-3-3ζ and the lysates of insect cells expressing human Chk1^111^. Kinase dead Chk1 and a truncated Chk1 construct lacking the C-terminal region (amino acids: 361–476) also interacted with 14-3-3ζ. Crucially, the Chk1 S345A mutant did not interact with 14-3-3ζ. 14-3-3σ binding to phosphorylated Chk1 was observed in co-IP experiments using HCT116 cells following radiation-induced DNA damage^112^. The interaction was not observed when phosphorylation of Chk1 was inhibited by treatment with UCN-01 (ref. ^112^). Chk1 has also been shown to associate with the fission yeast 14-3-3 proteins, Rad24 and Rad25, in a phosphorylation-dependent manner^51,113^. As demonstrated with human Chk1, Chk1 S345A mutation antagonises its association with Rad24 (ref. ^114^). These data, therefore, indicate that phosphorylation of Chk1 S345 residue within the SQ cluster is essential for 14-3-3ζ binding (Fig. 5a).

The S345 residue is located within a region (QGISFpSQPTC) that resembles a mode 2 14-3-3-binding motif, although it lacks the −4 positively charged residue relative to phosphorylated serine. Instead, a polar glutamine residue lies at the −5 position (Fig. 5a). Interestingly, the S317 residue is located within a region that closely resembles a mode I 14-3-3-binding motif. In this case, it has a −4 lysine residue (VKYSSpSQPEP) rather than a −3 arginine residue as found in classical mode 1 14-3-3-binding motifs (RSXpS/TxP) (Fig. 5a). There is no experimental evidence to suggest that phosphorylation of this residue plays a role in 14-3-3 binding; however, its proximity to S345 means that a bivalent interaction of phosphorylated Chk1 with a single 14-3-3 dimer is a strong possibility.

#### Putative role of the 14-3-3/Chk1 PPI in p53 regulation

Conclusive evidence that the 14-3-3/Chk1 PPI directly regulates p53 activity is currently lacking. However, Chk1 S345 is proximal to a nuclear export signal. S345A mutation leads to increased nuclear export as shown by fluorescence microscopy of HeLa cells expressing EYFP-labelled protein constructs^111^. Therefore, 14-3-3 proteins may play a role in retaining Chk1 in the nucleus, for example by antagonising Chk1 nuclear export mediated by Crm-1 protein (Fig. 5e)^111^. This is supported by immunofluorescence data from experiments conducted in yeast cells that show Chk1 nuclear accumulation following DNA damage is dependent on Rad24 expression^113^. Based on this evidence, it is reasonable to hypothesise that the 14-3-3/Chk1 PPI potentiates p53 phosphorylation and activation in the nucleus (Fig. 5e). In addition, the co-precipitation of Chk1 and p53 was shown to be dependent on the presence of 14-3-3σ. This suggests that 14-3-3σ plays a role in Chk1/p53 binding and thus potentially promotes p53 phosphorylation via this mechanism too^112^.

### 14-3-3 regulation of Bruton’s tyrosine kinase (BTK)

BTK is a non-receptor tyrosine kinase that plays a key role in B cell receptor signalling^115^. BTK upregulation has been demonstrated in various B cell malignancies such as chronic lymphocytic leukaemia and multiple myeloma; this has prompted the development of small molecule BTK inhibitors for the treatment of these diseases^116,117^. BTK has also been shown to have a pro-apoptotic and tumour suppressive function, which is mediated, in part, through its regulation of p53 (refs. ^118,119^) and p73 (ref. ^120^), showing that BTK can have antagonistic functions in cancer depending on the context^121^. BTK phosphorylates p53 at S15 and this promotes p53 stabilisation and a subsequent increase in p53 levels^118^. Furthermore, BTK has been shown to promote the upregulation of p53 target genes in HCT116 cells following doxorubicin induced DNA damage^118,119^. BTK also phosphorylates MDM2, which alleviates its negative effect on p53 activity^119^. Moreover, in a mouse model of p53-mediated accelerated ageing inhibition of BTK resulted in increased health-span and lifespan, supporting the important role of BTK in enhancing p53 activity^122^.

#### 14-3-3 binding mode

14-3-3ζ interacts with BTK in response to Akt-mediated phosphorylation of BTK at S51 and T495 located within the PH domain and the kinase domain respectively (Fig. 5b)^123^. IP and WB experiments conducted using primary human peripheral blood mononuclear cells identified an interaction between endogenous BTK and 14-3-3ζ. These results confirmed mass spectrometry proteomic analysis data of hematopoietic cell lines that identified 14-3-3ζ as a binding partner of BTK^123^. An interaction between BTK and GFP-labelled 14-3-3ζ was also observed in co-transfected COS-7 cells through IP and WB experiments^123^. The interaction was not observed in the presence of the 14-3-3 inhibitor BV02 (ref. ^123^).

Double alanine mutation of BTK S51A/T495A significantly diminished 14-3-3ζ binding as shown by IP/WB analysis. Furthermore, mutation of either residue of BTK S51A or T495A negatively affected 14-3-3ζ, but to a lesser extent when compared with double mutation^123^. This is another example that suggests a bivalent interaction involving distal phosphorylated motifs with a 14-3-3 dimer (c.f. HDAC4 and HDAC5). The sequence surrounding BTK T495 closely resembles a mode 2 14-3-3 binding motif (RHRFQpTQQLL), although it lacks the +2 proline residue (Fig. 5b). In addition, the phosphorylated S51 residue is located within a region of the protein that somewhat resembles a classical mode I 14-3-3-binding motif (RGRRGpSKKGS) (Fig. 5b).

#### The putative role of the 14-3-3/BTK PPI in p53 regulation

There is no direct evidence to confirm that the 14-3-3ζ/BTK PPI influences p53 activity. However, the binding of 14-3-3ζ to BTK promotes its nuclear export (Fig. 5e)^123^. siRNA knock-out of 14-3-3ζ protein in Namalwa B cells led to a marked increase in BTK nuclear accumulation as measured by WB analysis following cell fractionation^123^. There is also evidence that 14-3-3ζ promotes BTK ubiquitination and degradation (Fig. 5e)^123^. Expression of a double BTK mutant (S51A/T495A) in COS-7 cells led to reduced levels of ubiquitinated BTK as shown by IP using anti-ubiquitin antibodies^123^. Based on this evidence it is therefore likely that the 14-3-3ζ/BTK likely impedes BTK-mediated p53 phosphorylation and stabilisation.

### 14-3-3 regulation of ASK1

Apoptosis signal-regulating kinase-1 (ASK1) activates the p38 and JNK signalling pathways in response to oxidative stress^124,125^. p53 is phosphorylated by activated JNK and p38 kinases, which increases p53 stability and transcriptional activity and induces apoptosis^126,127^. Therefore, ASK1 has an indirect upstream role in p53 regulation.

#### 14-3-3 binding mode

14-3-3ζ binds to ASK1 in a manner dependent on the AKT-mediated phosphorylation of ASK1 S967, as shown by IP experiments conducted using MEF cells^128^. Mutation of ASK1 (S967A) disrupted the 14-3-3ζ/ASK1 PPI while the treatment of cells with a phosphatase inhibitor enhanced the interaction^129^. S967 is located within a classical mode 1 14-3-3-binding motif (YLRSIpSLPVP) (Fig. 5c), but no detailed investigation to characterise the PPI has been conducted^129^.

#### Putative role of the 14-3-3/ASK1 PPI in p53 regulation

Although there is no evidence that the 14-3-3ζ/ASK1 PPI directly regulates p53 activity, it does play a key role in controlling ASK1 activity, and thus p38 and JNK signalling pathways. Thus, it is likely that the PPI indirectly influences p53 (Fig. 5e). 14-3-3ζ inhibits ASK1 kinase activity and the subsequent activation of p38 and JNK^129–132^. Disruption of the 14-3-3ζ/ASK1 PPI by transfection of an ASK1 S967A mutant enhanced cell death in HeLa, COS-7 and 393T cells^129^. Overexpression of 14-3-3ζ suppressed ASK1-mediated apoptosis^129^. During high levels of oxidative stress, ASK1 is dephosphorylated at pS967 by PP1/PP2A-like phosphatase, which results in the loss of 14-3-3ζ binding and the subsequent activation of the p38 and JNK MAPK signalling pathways^131^. This was shown in COS-7 cells whereby treatment with H_2_O_2_ led to increased ASK1, p38 and JNK kinase activity^131^.

### 14-3-3 regulation of AKT

AKT kinase phosphorylates MDM2, promoting its nuclear localisation and association with p53 (refs. ^41,42^). Thus, it is another an example of a kinase with an indirect upstream role in p53 regulation.

#### 14-3-3 binding mode

14-3-3σ has been shown to associate with AKT in A459 cells in response to DNA damage as shown by IP experiments^37^. The interaction 14-3-3σ AKT involves the CTD of 14-3-3σ (amino acid residues: 152–248) and the kinase domain of AKT (amino acids: 1–147) (Fig. 5d), as shown by co-IP experiments using deletion mutants of 14-3-3σ and AKT^37^. This is another example of a PPI relying on a non-canonical interaction with 14-3-3σ.

#### Putative role of the 14-3-3/AKT PPI in p53 regulation

14-3-3σ inhibits AKT kinase activity^37^. Therefore, although there is no direct evidence, it is likely that the 14-3-3/AKT PPI contributes to p53 activation (Fig. 5e). In R1B/L17 cells, the expression of 14-3-3σ impairs the kinase activity of AKT for its substrate GSK3β^37^ 14-3-3σ is not a substrate for AKT and therefore is not a competitive inhibitor of AKT kinase^37^. Furthermore, AKT activation is increased in 14-3-3σ-deficient HCT116 cells^37^. 14-3-3σ also inhibits the AKT-mediated survival of cancer cells. Transfection of a 14-3-3σ adenovirus into Rat1-AKT cells, which have constitutive AKT expression, induces apoptosis^37^. Transfection of the 14-3-3σ CTD (amino acid residues: 152–248) was as effective at inducing apoptosis as wt 14-3-3σ further confirming the involvement of a non-canonical 14-3-3 PPI^37^.

## Conclusions and perspectives

Realising the vast genetic heterogeneity of cancers, as well as the frequent emergence of resistance, has forced a change in the approach to antineoplastic drug discovery^133^. Single-target therapies that inhibit mutated oncogenes, which have been a staple of twenty-first century treatments, have proved to be insufficient to eliminate most cancers, and now we better understand the reasons behind this problem. Strategies that could attack the root changes present in all the cells of a given tumour are currently being hailed as promising alternatives, although they so far have proved elusive. A common feature of cancer cells is the inactivation of the p53 pathway at some point of the neoplastic development, which only in half of the cases is due to mutations in p53 itself. Thus, reactivating p53 in the cells that have the wild-type form could potentially trigger an apoptotic response to which many tumours would be responsive. However, achieving this goal has been difficult. For example, the Nutlin compound class effectively inhibits MDM2-mediated ubiquitination and degradation of p53 (ref. ^134^), but have not been able to progress through clinical trials. We propose that 14-3-3 proteins could be alternative targets to the reactivation of p53, due to their extensive role in direct or indirect regulation of the pathway.

Here, we have highlighted the different effects of the 14-3-3 isoforms on regulators of p53 (Table 2). Within this network, 14-3-3 proteins predominantly regulate the subcellular localisation of the interacting protein partner, but also facilitate structural changes, influence interactions with other proteins or modulate their function. Their final effects on ubiquitination, acetylation, phosphorylation and p53 itself describe a pleiotropic pattern of activity that could be regulated to achieve the desired effects. For instance, new small molecules could be designed to stabilise the PPI between different 14-3-3 isoforms and p53, MDM2, MDMX, COP1, HDAC5, CHK1 or AKT, which would all result in increased p53 activity. Compounds that have some of these effects, such as the ‘molecular glue' Fusicoccin A, have already been identified^23^. Conversely, we could seek to inhibit the PPI between 14-3-3 and SIRT2, HDAC4, BTK or ASK1, which would also lead to p53 reactivation. Many of the proposed impacts of 14-3-3 proteins on the p53 pathway are just being proposed and will require more research to be confirmed before they can be considered potential therapeutic targets. Others have already been extensively characterised and are being considered for drug development. In particular, 14-3-3σ has distinctive functions and several of its interactions are not phosphorylation dependent but rely on a hitherto poorly understood secondary binding site.

**Table 2:** A summary of the 14-3-3 PPIs that are directly implicated or have a putative role in the regulation of p53, and the desired strategy for developing novel antineoplastic therapies.

14-3-3-binding partner	14-3-3 isoform	Binding mode	p53 regulation	Therapeutic strategy	Review (key refs.)
**Direct link between 14-3-3 PPI and p53 regulation**					
p53	γ	Phosphorylation dependent (pS366, pS378, pT387; monovalent)	•Increases p53 transcriptional activity	Stabilisation (e.g. FC-A)	2.1 (12–19)
	ε		•Enhances p53 binding to DNA •Increases p53 transcriptional activity		
	ζ				
	σ	Phosphorylation dependent (pS366, pS378, pT387; monovalent) Non-canonical interaction: 14-3-3 CTD (amino acids: 153–248)	•Increases p53 protein levels •Increases p53 transcriptional activity		2.2 (20–27)
	τ				
MDM2	σ	Phosphorylation dependent (unknown sites) Non-canonical interaction(s): MDM2 CTD (amino acids: 440–491) 14-3-3 CTD (amino acids: 153–248)	•Increases p53 protein levels •Increases p53 transcriptional activity	Stabilisation	3.1.1-2 (35–37)
	(α)β, γ, ε, η, ζ, τ	Phosphorylation dependent (pS166 and pS186; bivalent)	•Not known	?	3.1.3–4 (40)
MDMX	(α)β, γ, ε, η, ζ, τ	Phosphorylation dependent (pS342 and pS367; bivalent)	•Increases p53 protein levels	Stabilisation	3.2 (55, 57-62)
COP1	σ	Phosphorylation dependent (pS387; monovalent)	•Increases p53 protein levels •Increases p53 transcriptional activity •Inhibits p53 nuclear export	Stabilisation	3.3 (63–65)
SIRT2	(α)β, γ	Phosphorylation dependent (putative site: pS368; monovalent)	•Decreases p53 transcriptional activity	Inhibition	4.1 (82)
**No direct link between 14-3-3 PPI and p53 regulation**					
HDAC4	(α)β, ε	Phosphorylation dependent (pS246, pS467, pS632; bivalent)	•HDAC4 cytoplasmic sequestration •Putative: Increases transcription of p53 pro-survival target genes	Inhibition	4.2 (87–89)
HDAC5	(α)β, ε	Phosphorylation dependent (pS259, pS498, pS661; bivalent?)	•HDAC5 nuclear exclusion and cytoplasmic sequestration •Putative: Inhibits p53 acetylation (K120) and increases transcription of pro-apoptotic genes	Stabilisation	4.3 (87–89)
HDAC7	ε	Phosphorylation dependent (pS155, pS358, pS486; monovalent)	•Increases HDAC7 levels but promotes cytoplasmic accumulation. •Implications for p53 regulation are unclear	?	4.4 (91, 95–96)
Chk1	(α)β, ζ, σ	Phosphorylation dependent (pS345; monovalent?)	•Chk1 nuclear accumulation •Putative: promotes p53 phosphorylation and stabilisation	Stabilisation	5.1 (111–114)
BTK	ζ	Phosphorylation dependent (pS51, pT495; bivalent)	•BTK nuclear exclusion and ubiquitination •Putative: prevents BTK-mediated p53 phosphorylation and stabilisation	Inhibition	5.2 (123)
ASK1	ζ	Phosphorylation dependent (pS966; monovalent)	•ASK1 inhibition. •Putative: prevents p53 activation via the p38/JNK pathways	Inhibition	5.3 (128–129)
AKT	σ	Non-canonical interaction: 14-3-3 CTD (amino acids: 1–147)	•AKT inhibition •Putative: stabilises p53 by preventing MDM2 phosphorylation.	Stabilisation	5.4 (37)

Our review has focused on the nature of the PPI that would need to be enhanced or disrupted in order to achieve p53 reactivation, including its putative site when known. We have also highlighted the distinction between phosphorylation-dependent interactions that are monovalent and those that are bivalent and dependent on 14-3-3 dimerisation. These are important subtleties that may provide new avenues for the development of chemical modulators of 14-3-3 interactions. Molecular tools that facilitate more detailed and expansive studies into the role of 14-3-3 in p53 activation are urgently needed. These will enhance our fundamental understanding of the complex regulatory network and pave the way for the development of selective and potent antineoplastic drugs that could exploit a hidden weakness that lies at the core of at least half of all cancer cells.
